# Inevitable epistemological conflict: Reflections on a disagreement over the relationship between science and indigenous and local knowledge

**DOI:** 10.1007/s13280-022-01739-7

**Published:** 2022-05-17

**Authors:** Maris Boyd Gillette, Benedict E. Singleton

**Affiliations:** Environmental Social Science, School of Global Studies, University of Gothenburg, Gothenburg, Sweden

Comment to: Reyes-García, V., Á. Fernández-Llamazares, Y. Aumeeruddy-Thomas, P. Benyei, R.W. Bussmann, S.K. Diamond, D. García-del-Amo, S. Guadilla-Sáez, et al. 2022a. Recognizing Indigenous peoples' and local communities' rights and agency in the post-2020 Biodiversity Agenda. *Ambio* 51: 84–92. https://doi.org/10.1007/s13280-021-01561-7

Lopez-Maldonado, Y. 2022. Practice what you preach: Ensuring scientific spheres integrate Indigenous Peoples’ and Local Communities’ right and agency too. *Ambio*. 51: 811–812. https://doi.org/10.1007/s13280-021-01663-2

Reyes-Garcia, V., Á. Fernandez-Llamazares, Y. Aumeeruddy-Thomas, P. Benyei, R.W. Bussmann, D. Garcia-Del-Amo, N. Hanazaki, A.C. Luz, et al. 2022b. Response to “Practice what you preach: Ensuring scientific spheres integrate Indigenous Peoples’ and Local Communities’ rights and agency too” by Lopez-Maldonado. *Ambio* 51: 813–814. https://doi.org/10.1007/s13280-021-01676-x

In the recent exchange between Reyes-García et al. and Lopez-Maldonado, important points are raised, but both sides fail to recognise their embeddedness in an ongoing conflict about the epistemological boundaries of science and the social category of expert within the “extended peer community” (Funtowicz and Ravetz 1994). In the following paragraphs, we briefly discuss what we feel has been missed, arguing for an approach to western science-ILK engagements called “productive complicity” (Singleton et al. 2021).

In their initial paper, Reyes-García et al. (2022a) argue for integrating indigenous and local knowledge (ILK) into biodiversity science, echoing other environmental scientific rhetoric (cf. Singleton et al. 2021). They argue for the importance of ILK for biodiversity and sustainability transitions and the importance for science-ILK integration for indigenous peoples and local communities (IPLC) rights and territorial claims. They assert that “rights and agency” must be foregrounded in collaborations between scientists and IPLC to co-produce “emergent knowledge that supports conservation” (86).

Lopez-Maldonado (2022) argues that ILK is “eroded” through engagements with western science, and criticises Reyes-Garcia et al. for inadequately recognising the colonial past and present of hegemonic western science. This renders knowledge co-production and systemic integration unreasonable. IPLC do not necessarily benefit from collaborating with scientists complicit in “colonizing methodologies and approaches” that miss “the essence of ILK” and constitute “an existential threat to humanity” (811). In their rejoinder, Reyes-García et al. acknowledge science’s relationship with imperialism, but assert Lopez-Maldonado depicts a strawman; that the history of efforts to integrate ILK into science and policy reveals efforts to learn from and ameliorate science’s colonialist tendencies (2022b). They emphasise the pragmatic value of their work as a call for improving respect for IPLC knowledge, rights and agency in exclusionary contexts (813).

Looking at this discussion, we see dissonance over the make-up of ILK and its relationship to science. To Reyes-García et al., ILK comprises a body of knowledge which contains information valuable to both science and society, which should, thus, be integrated and protected. ILK is “holistic”, having “multiple values”, and thus, a good source of environmental ethics (2022a, p. 86). However, many of their examples of IPLC-scientist collaborations actually describe IPLC gathering data useful to western science, rather than autonomous IPLC knowledge systems or processes. In their rejoinder (2022b), they acknowledge the contribution of indigenous scientists, while suggesting that even though IPLC explicitly have “essential knowledge” about environmental ethics, their main interest is ILK that “supports conservation” as practised by western biodiversity scientists.

In contrast, Lopez-Maldonado (2022) demarcates a clear boundary between ILK and western science. *Western* science is strongly complicit in the annihilation of vital human knowledge and distinctly separate from indigenous people (812). She also complains that Reyes-Garcia et al. are “reluctant to consider ILK as a proper science” and “still treat IPLC as illiterate or not being able to communicate fundamental knowledge of the Earth’s system function, despite IPLC hold their own methods and approaches for that” (812). In other words, ILK *should* be labelled *science* (2022).

In our view, this is a scientific boundary conflict regarding the construction and functioning of the extended peer community (Funtowicz and Ravetz 1994). Both sides agree that a category of expert knowledge called “science” has value, differing over rights to define its content and, thus, make expert decisions about biodiversity management. Arguments advocating for selective knowledge integration on the one hand (Reyes-García et al. 2022a, b), or knowledge separation on the other (Lopez-Maldonado 2022), are inadequate to capture the variety of interactions required for conservation to realise its full potential.

As we argue elsewhere, an alternative framing for scientist-IPLC engagements and indeed extended peer communities more generally is “productive complicity” (Singleton et al., 2021). Productive complicity refers to contingent, politically sensitive collaborations that affirm temporary but useful essentialisms, such as “indigenous and local knowledge”, in order to achieve shared objectives. Such partnerships consciously confront the contextual, temporal and political dimensions of any and all research collaboration. They build on researchers’ reflexivity or awareness of inherent essentialisms in situationally bounding knowledge as “science” or non-science, and her/his/their own complicity in power relations. Productive complicity entails participants self-consciously and critically assessing the repercussions of particular acts of collaboration from a multitude of normative perspectives. It entails awareness of broader social-ecological contexts, not least in relation to dispossessed and marginalised groups. As a mode of partnering, this focuses attention on the dynamic, political and temporally situated nature of collaboration. It facilitates scientists and IPLC strategically channelling their partnerships toward the emancipation of marginalised communities and the pursuit of equitable environmental outcomes for humans and nonhumans.

New knowledge-making actors increasingly seek participation in the operations of environmental and conservation science. Who belongs to the extended peer community of these fields and where the boundaries are drawn for what is considered “science” are affected by this development. We encourage knowledge producers of all stripes to embrace this situation and develop creative methods for respectful and reflexive engagement. Recognising science-IPLC cooperation as acts of productive complicity is a useful first step.
